# Global Conformational Dynamics of a Y-Family DNA Polymerase during Catalysis

**DOI:** 10.1371/journal.pbio.1000225

**Published:** 2009-10-27

**Authors:** Cuiling Xu, Brian A. Maxwell, Jessica A. Brown, Likui Zhang, Zucai Suo

**Affiliations:** 1 Department of Biochemistry, The Ohio State University, Columbus, Ohio, United States of America; 2 Ohio State Biophysics Program, The Ohio State University, Columbus, Ohio, United States of America; 3 Ohio State Biochemistry Program, The Ohio State University, Columbus, Ohio, United States of America; 4 Molecular, Cellular, and Developmental Biology Program, The Ohio State University, Columbus, Ohio, United States of America; 5 Comprehensive Cancer Center, The Ohio State University, Columbus, Ohio, United States of America

## Abstract

Replicative DNA polymerases are stalled by damaged DNA while the newly discovered Y-family DNA polymerases are recruited to rescue these stalled replication forks, thereby enhancing cell survival. The Y-family DNA polymerases, characterized by low fidelity and processivity, are able to bypass different classes of DNA lesions. A variety of kinetic and structural studies have established a minimal reaction pathway common to all DNA polymerases, although the conformational intermediates are not well defined. Furthermore, the identification of the rate-limiting step of nucleotide incorporation catalyzed by any DNA polymerase has been a matter of long debate. By monitoring time-dependent fluorescence resonance energy transfer (FRET) signal changes at multiple sites in each domain and DNA during catalysis, we present here a real-time picture of the global conformational transitions of a model Y-family enzyme: DNA polymerase IV (Dpo4) from *Sulfolobus solfataricus*. Our results provide evidence for a hypothetical DNA translocation event followed by a rapid protein conformational change prior to catalysis and a subsequent slow, post-chemistry protein conformational change. Surprisingly, the DNA translocation step was induced by the binding of a correct nucleotide. Moreover, we have determined the directions, rates, and activation energy barriers of the protein conformational transitions, which indicated that the four domains of Dpo4 moved in a synchronized manner. These results showed conclusively that a pre-chemistry conformational change associated with domain movements was too fast to be the rate-limiting step. Rather, the rearrangement of active site residues limited the rate of correct nucleotide incorporation. Collectively, the conformational dynamics of Dpo4 offer insights into how the inter-domain movements are related to enzymatic function and their concerted interactions with other proteins at the replication fork.

## Abstract 2

High-resolution analysis of protein, and DNA conformational changes during DNA polymerization, established relationships between the enzymatic function and conformational dynamics of individual domains for a DNA polymerase.

## Author Summary

Faithful replication of genomic DNA by DNA polymerases is crucial for maintaining the genetic integrity of an organism. If DNA becomes damaged, specialized lesion-bypass DNA polymerases are recruited to correct errors in the DNA. A variety of kinetic and structural studies have established a minimal kinetic mechanism common to all DNA polymerases. This mechanism includes several steps involving discrete protein conformational changes. However, the inter-relationship between conformational dynamics and enzymatic function has remained unclear, and identification of the rate-limiting step during nucleotide incorporation has been controversial. In this study, we monitored the directions and rates of motion of domains of a lesion-bypass polymerase during correct nucleotide incorporation. Our study provides several significant findings. First, the binding of a correct nucleotide induces a fast and surprising DNA translocation event. Second, all four domains of the polymerase rapidly move in a synchronized manner before and after the polymerization reaction. Third, repositioning of active site residues is the rate-limiting step during correct nucleotide incorporation. Thus, the motions of the polymerase and the polymerase-bound DNA substrate are tightly coupled to catalysis.

## Introduction

Elucidating the mechanism of enzyme catalysis encompasses the identification and characterization of each chemical and conformational intermediate occurring along the reaction pathway [1]. Among the six families (A, B, C, D, X, and Y) of DNA polymerases, crystallographic studies have captured these enzymes, which exhibit a similar three-dimensional right hand shape composed of the finger, palm, and thumb domains, in various states. By superimposing these structural snapshots during a catalytic cycle, conformational changes have been revealed as the polymerase sequentially binds the DNA and nucleotide substrates. In general, nucleotide binding induces a significant structural change involving an open-to-close transition of the finger domain for the A-, B-, and some X-family DNA polymerases [2]–[6] while ternary complex formation for the Y- and some X-family members [7],[8] leads to the subtle repositioning of select active site residues. The open-to-close finger domain transition induced by nucleotide binding provides the basis for an induced-fit model, which has been proposed to correspond to the rate-limiting step of correct nucleotide incorporation. Numerous stopped-flow studies monitoring a single fluorophore, either on DNA (e.g., 2-aminopurine) [9]–[13] or on the finger domain (tryptophan or fluorescent dye) of a DNA polymerase [14],[15], have generated interesting but contradictory evidence for this assignment because the fluorescence intensity of a fluorophore can be affected by many factors, thereby complicating data interpretation. Recently, this assignment of the rate-limiting step has been forcefully questioned due to fluorescence resonance energy transfer (FRET)-based evidence for two A-family DNA polymerases [16]–[18], which shows that the closure rate of the finger domain is too fast to limit correct nucleotide incorporation. Therefore, it has been hypothesized by us [19],[20] and others [16]–[18],[21] that the rate-limiting step corresponds to the subtle repositioning of active site residues, which are critical for properly aligning two magnesium ions, the 3′-hydroxyl of the primer terminus, the α-phosphate of the incoming dNTP, and the conserved carboxylate residues in the active site.

**Figure 1:**
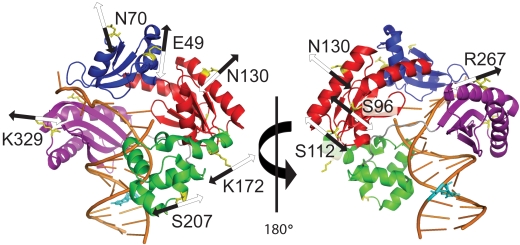
Front and back views of domain motions during a single, correct nucleotide incorporation. The domains of Dpo4 are shown in blue (finger), red (palm), green (thumb), and purple (LF); the DNA is in gold; all of the nine mutant residues (Table S1) are in yellow and the Alexa488-labeled DNA base is in cyan. The arrows represent the direction of residue movement based on the FRET signals for phase P_1_ (black) and phase P_2_ (white).

To the best of our knowledge, no studies have characterized the global conformational dynamics of a DNA polymerase undergoing catalysis. Besides the finger domain, other core domains of a DNA polymerase may undergo significant structural changes and movements during nucleotide incorporation. To establish a better understanding of the interrelationship between protein conformational dynamics and nucleotide incorporation, we chose to investigate Dpo4, a 40 kDa Y-family DNA polymerase containing no tryptophan residues and a single cysteine. In addition to the three aforementioned polymerase core domains, Dpo4 also possesses a little finger (LF) domain that is unique to the Y-family DNA polymerases (Figure 1) [22]–[24]. After generating two FRET systems (i.e., donor on DNA/acceptor on each domain of Dpo4 and donor on the LF domain/acceptor on the finger domain) using protein engineering methods, we monitored time-dependent FRET signal changes during a single, correct nucleotide incorporation in order to probe how each domain of Dpo4 moved relative to either DNA or LF in real time. We observed a surprising DNA translocation event induced by nucleotide binding and concerted motions of all four of Dpo4's domains during catalysis. We also conclusively excluded rapid domain closure as the rate-limiting step of the kinetic mechanism for correct nucleotide incorporation.

## Results/Discussion

### Design of Two FRET Systems

Recently, our crystallographic study of Dpo4 reports that, upon nucleotide binding, no large-scale domain movements are observed, but local conformational changes occur for active site residues (Y10, Y48, R51, and K159) near the nucleotide binding pocket [8]. To examine if these crystallographic observations are true in solution, we investigated the conformational changes of Dpo4 during a single, correct nucleotide incorporation by monitoring the real-time FRET changes with a stopped-flow apparatus. Conformational changes were detected using two FRET systems, which monitored (i) the motions of specific residues on each domain relative to the enzyme-bound DNA substrate and (ii) the motions of the finger domain relative to the LF domain. For system (i), the FRET pair consisted of an Alexa488 donor fluorophore covalently linked to the ninth primer base [22] from the primer 3′-terminus in S-1 or S-2 DNA (Table 1) and an Alexa594 acceptor fluorophore on a site-specific, substituted cysteine, which was not a functionally conserved residue in Dpo4 (Figure 1 and Table S1). At least one α helix residue and one loop residue in each domain were selected for attaching Alexa594 (Table S1) in order to exclude the effect of protein secondary structure on the observed real-time FRET. For system (ii), an intrinsic tryptophan donor (Y274W) was engineered into the LF and the 7-diethylamino-3-(4′-maleimidylphenyl)-4-methylcoumarin (CPM) acceptor fluorophore was attached to a single cysteine mutation in a loop of the finger domain (Table S2). The Förster radii (R_0_) of the FRET pairs of Alexa488/Alexa594 and tryptophan/CPM are 60 and 30 Å, respectively [25]. Notably, the reason why these two FRET systems can be established through protein engineering methods is because Dpo4 contains no native tryptophan residues and only one native cysteine residue. This native cysteine residue was mutated to serine so that only a single cysteine was labeled with either Alexa594 or CPM (Materials and Methods). DNA polymerase activity of each fluorophore-labeled and unlabeled Dpo4 mutant was measured under single-turnover conditions using radioactive chemical-quench techniques; these rates were determined at both 20°C and 37°C (Table S3). Relative to wild-type Dpo4, the observed rate constants (*k_obs_*) indicated that the mutants, with or without the dye, were catalytically active. Furthermore, the circular dichroism spectra of unlabeled mutants were nearly identical to wild-type Dpo4 (Figure S1), thereby indicating these point mutations did not significantly alter the enzyme's secondary structure.

**Table 1:** Sequences of DNA substrates.

Substrate	DNA Sequence
S-1	5′-CG AGC CGT CGC A**T**C CTA CCG C-3′
	3′-GC TCG GCA GCG TAG GAT GGC GAC GTC GTA G-5′
S-2	5′-CG AGC CGT CGC A**T**C CTA CCG ***C***-3′
	3′-GC TCG GCA GCG TAG GAT GGC GAC GTC GTA G-5′
S-3	5′-CG AGC CGT CGC ATC CTA CCG C-3′
	3′-GC TCG GCA GCG TAG GAT GGC GAC GTC GTA G-5′
S-4	5′-CG AGC CGT CGC ATC CTA CCG ***C***-3′
	3′-GC TCG GCA GCG TAG GAT GGC GAC GTC GTA G-5′

*Note*: **T** and *C* denote Alexa488-attached to a 5-C6-Amino-2′-deoxythymidine and 2′,3′-dideoxycytidine, respectively.

### FRET Changes Induced by Association between Dpo4 and Substrates

**Figure 2:**
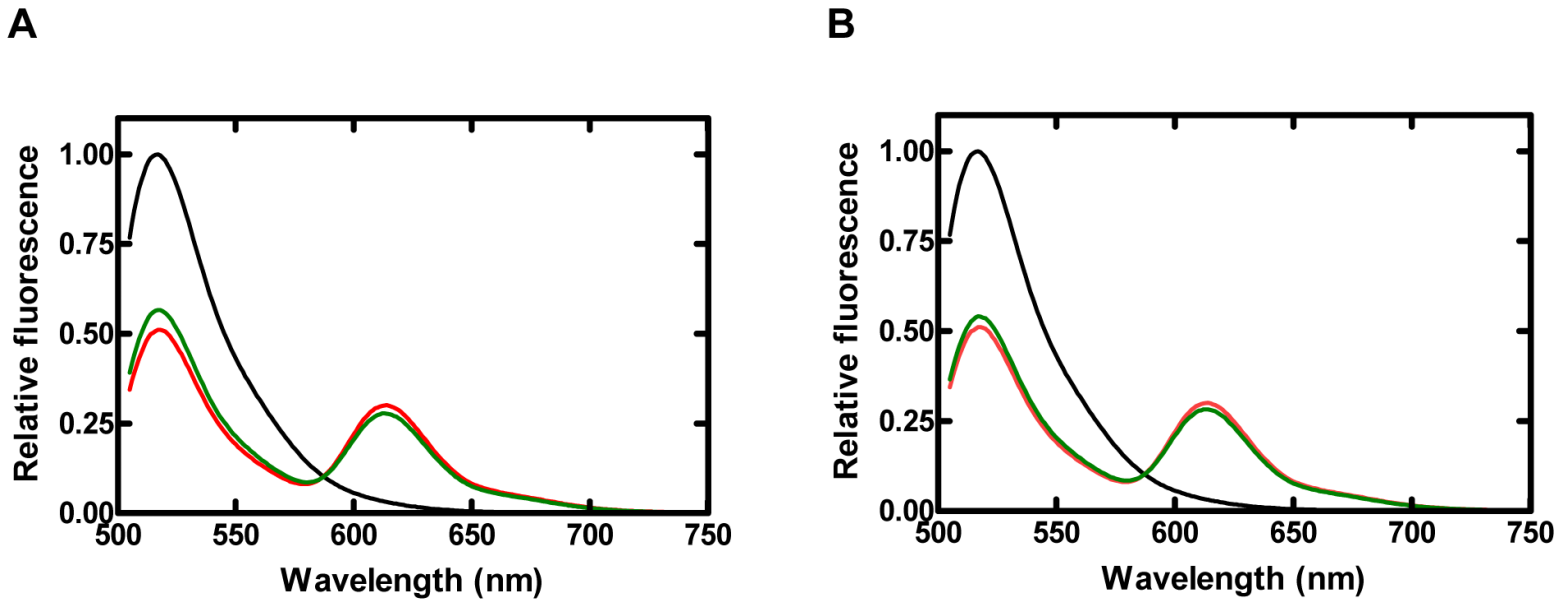
Steady-state fluorescence spectra of finger domain mutant (N70C) at 20°C. Alexa488-labeled DNA (100 nM, black trace) was excited at a wavelength of 493 nm. The sequential addition of Alexa594-labeled Dpo4 (600 nM) and dTTP (1 mM) produced the red and green traces, respectively. Spectra were normalized to 1 by using the donor as a reference. Emission spectra are shown for both (A) S-1 and (B) S-2 DNA substrates.

To verify the conformational changes were related to a FRET signal, steady-state fluorescent assays were employed using Dpo4 mutants labeled with Alexa594, either S-1 or S-2 DNA substrates attached to Alexa488, and the correct nucleotide, dTTP. First, control experiments were performed with either labeled protein binding to unlabeled DNA or unlabeled protein binding to labeled DNA in the presence or absence of dTTP at 20°C. Although Dpo4 binds to DNA tightly with an affinity of 3–10 nM [19],[26], the emission spectra for these control experiments did not show any significant fluorescence changes of FRET (Figure S2A and S2B). In contrast, addition of the labeled Dpo4 N70C mutant to the labeled DNA alone (black trace) resulted in a large reduction in donor (Alexa488) fluorescence accompanied by a concomitant increase in acceptor (Alexa594) fluorescence (red trace) upon exciting at the donor excitation wavelength of 493 nm (Figure 2). The dramatic acceptor fluorescence increase was likely due to efficient FRET between donor and acceptor. After the addition of 1 mM correct incoming nucleotide dTTP to the Dpo4•DNA (S-1 or S-2) complex, a decrease in FRET (green trace) was observed (Figure 2) as indicated by an increase in donor fluorescence and a decrease in acceptor fluorescence. The changes in both donor and acceptor fluorescence were slightly larger with S-1 than with S-2 when superimposing their steady-state fluorescence spectra (unpublished data). Although the amplitude of the FRET change induced by dTTP addition was relatively small, the experimental result was reproducible. Similar phenomena were observed for the LF, palm, and thumb domain mutants (unpublished data). The FRET signal represented conformational changes that may be predominantly pre-catalytic, since a similar trend was detected with dideoxy-terminated S-2 DNA (Figure 2B). Overall, these results confirmed that this FRET system indeed monitored Dpo4's conformational transitions during the nucleotide incorporation cycle.

### DNA Sliding, Synchronized Intra-Domain Rotation, and Inter-Domain Motions Relative to DNA during Catalysis

**Figure 3:**
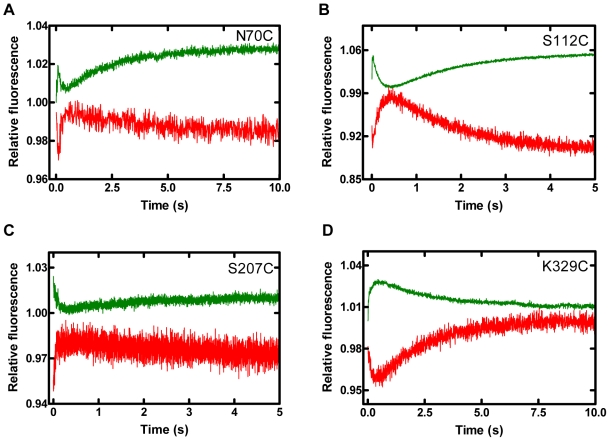
Stopped-flow kinetics of dTTP incorporation into S-1 DNA at 20°C. Dpo4 mutant•S-1 DNA complexes were reacted with dTTP and fluorescence was monitored using a stopped-flow apparatus. Donor (green) and acceptor (red) traces are shown for (A) the finger (N70C), (B) palm (S112C), (C) thumb (S207C), and (D) LF (K329C) domains. Data for finger (E49C), palm (S96C and N130C), thumb (K172C), and LF (R267C) residues are shown in Figure S3. Each Dpo4 mutant (Table S1) and S-1 were labeled with Alexa594 and Alexa488, respectively. Notably, some changes in fluorescence upon dTTP binding occurred during the instrument's dead time and the donor and acceptor fluorescence signals at time zero or close to time zero were not recorded.

Real-time kinetic FRET experiments were performed to further dissect the FRET change in Figure 2 and to measure the conformational transition rates of the nucleotide-induced domain movements for the binary Dpo4•DNA (S-1 or S-2) complex at 20°C and 37°C. First, domain motions relative to DNA were investigated. No time-dependent fluorescence change was detected in control experiments, which were performed as stated above (Figure S2C). Upon the addition of dTTP to the labeled Dpo4•S-1 complex, certain residues on the finger (N70C) and palm (S112C and N130C) domains exhibited three FRET phases while all other mutants showed two phases (Figure 3 and Figure S3). As expected, the time-dependent FRET signal changes of acceptor were correlated with the fluorescence signal changes of the donor, and hereafter only the acceptor signal is discussed. For the finger (N70C) and palm (S112C and N130C) domain mutants, the three phases were defined by an initial, rapid FRET decrease phase (P_0_) followed by a second, fast increase phase (P_1_) and a third, slow decrease phase (P_2_) (Figure 3A, 3B, and Figure S3C). Any change in FRET represents a change in the distance between two fluorophores, which subsequently indicates the motion of a Dpo4 domain relative to DNA. Thus, the above-mentioned FRET changes indicated a rapid DNA translocation event during P_0_ (see below), closure of the finger and palm domains to grip the DNA substrate during P_1_, and reopening of these two domains during P_2_ (Figure 1). Meanwhile, the remaining mutants showed two phases that were similar to the aforementioned P_1_ and P_2_ phases (Figure 3C, 3D, and Figure S3). However, depending upon the mutant, the directionality of these two phases' FRET signals varied. The FRET traces for the thumb mutants (K172C and S207C) exhibited a “gripping-reopening” motion analogous to P_1_ and P_2_ for N70C, S112C, and N130C (Figure 3 and Figure S3). In contrast, residues in the LF (R267C and K329C), finger (E49C), and palm (S96C) domains moved away and then towards the DNA (Figure 3D and Figure S3).

To determine whether these domain movements for each phase were synchronized, we fit each individual phase to a single-exponential equation in order to extract the rates of the conformational transitions (Table S4). Interestingly, an initial, rapid FRET decrease, P_0_, for the finger (N70C) and palm (S112C and N130C) residues was detected, which suggested a DNA translocation event that increased the distance between the FRET pair. This event was likely through the rotation of the DNA duplex and can also be inferred from the superimposition of our published binary and ternary crystal structures of Dpo4 [8]. Interestingly, a stopped-flow study of *S. acidocaldarius* DinB homolog (Dbh), a Y-family homolog of Dpo4, has also inferred a similar DNA translocation event based on real-time fluorescence changes of a single fluorophore (2-aminopurine) in DNA [9], although the evidence is indirect and questionable. Unfortunately, the rate of P_0_ occurred too fast to be determined accurately and is not reported here. Since this P_0_ phase occurred near the time resolution of our instrument, the corresponding FRET decrease could not be distinguished when P_1_ also resulted in a FRET decrease as with residues on the LF, E49C on the finger, and S96C on the palm. Additionally, the distances between the residues on the thumb domain and the labeled DNA base were approximately perpendicular to the direction of DNA translocation in P_0_. Therefore, the change in distance for each of these events likely produced a change in fluorescence below the level of sensitivity of our system. Interestingly, the rates for each domain during P_1_ and P_2_ were similar at each reaction temperature for both the donor and acceptor fluorescence traces, and so the average rates of P_1_ and P_2_ are used to simplify the discussion in the later section. However, the average P_2_ rate was approximately 25- or 5-fold slower at 20°C and 37°C, respectively, than that of P_1_ (Table S4). The similar rates of the donor and acceptor further confirmed that the observed fluorescence changes were due to a time-dependent FRET process. Based on the sites tested herein, the domains of Dpo4 moved in a concerted motion upon binding a correct nucleotide.

However, the relative direction of residues within each domain was not always identical, which likely reflects the rotational nature of the polymerase core domains assembling the active site for catalysis. Consistently, neutron spin-echo spectroscopic studies of *Thermus aquaticus* DNA polymerase reveal that this A-family enzyme does not function as a rigid body in solution but uses coupled inter-domain motions and intra-domain rotations to coordinate catalysis [27]. After examining the ternary crystal structure of Dpo4 [22], a rotational axis in the palm domain between β-sheets 5 and 6, where the active site is in closer proximity to the bound DNA and dNTP, would be consistent with an inward motion of residues S112C and N130C and an outward motion of residue S96C. Similarly, for the finger mutants N70C and E49C, a rotational motion about an axis between α-helixes B and C would allow the finger domain to be in greater contact with the substrates despite the anomalous directionality of the FRET traces. The LF and thumb domains may rotate upon formation of a ternary complex, although the locations examined in this study did not confirm this possibility.

### Conformational Transitions in P_2_ Occurred after Phosphodiester Bond Formation

**Figure 4:**
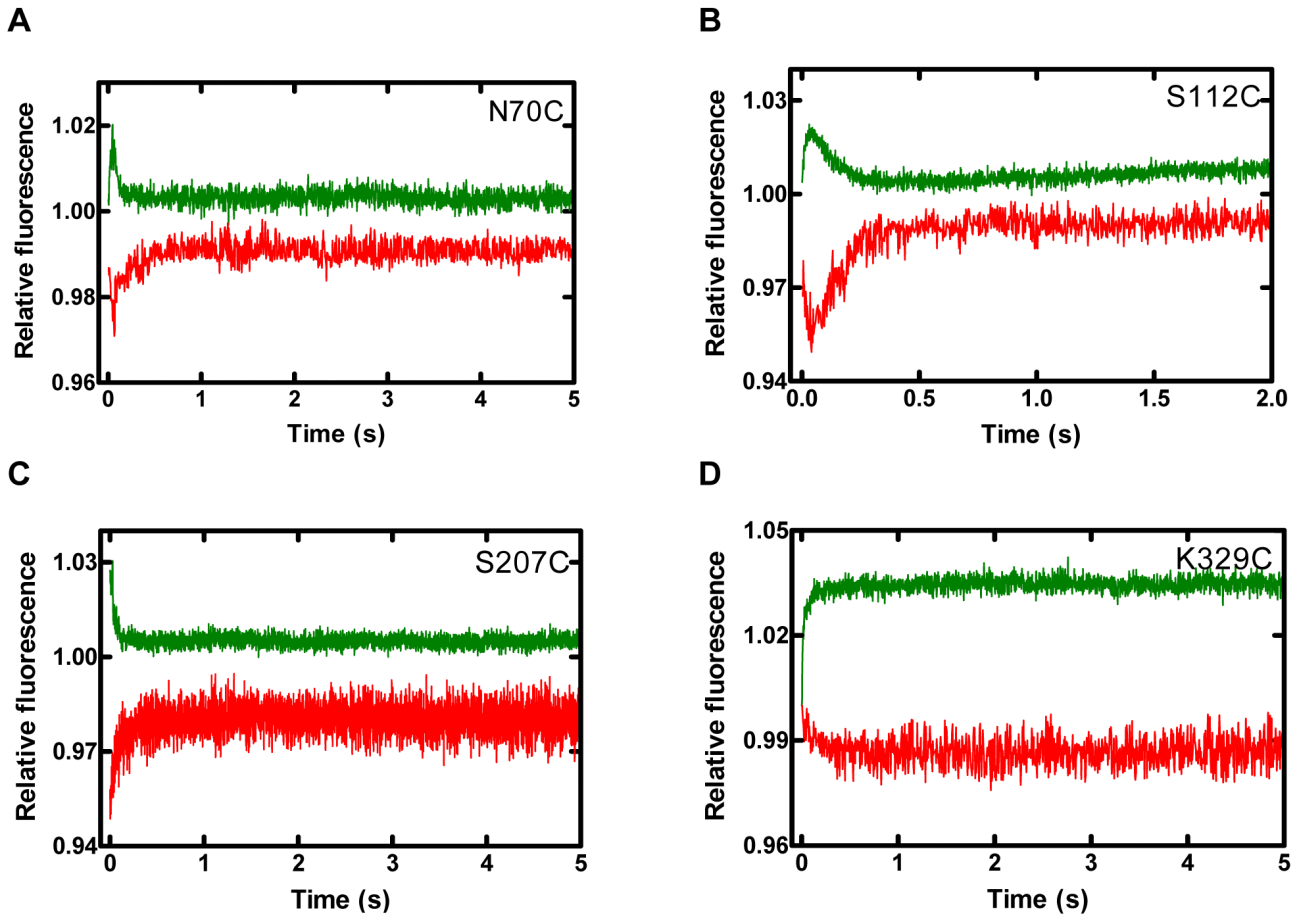
Stopped-flow kinetics of dTTP incorporation into S-2 DNA at 20°C. Dpo4 mutant•S-2 DNA complexes were reacted with dTTP and the fluorescence was monitored using a stopped-flow apparatus. Donor (green) and acceptor (red) traces are shown for the (A) finger (N70C), (B) palm (S112C), (C) thumb (S207C), and (D) LF (K329C) domains. Data for finger (E49C), palm (S96C and N130C), thumb (K172C), and LF (R267C) residues are shown in Figure S4. Each Dpo4 mutant (Table S1) and S-2 were labeled with Alexa594 and Alexa488, respectively. Notably, some changes in fluorescence upon dTTP binding occurred during the instrument's dead time and the donor and acceptor fluorescence signals at time zero or close to time zero were not recorded.

Our next objective was to discern if these conformational changes were occurring before or after the chemistry step. Using a non-extendable S-2 DNA substrate, we observed that P_2_ for all mutants was absent while the other phases remained unchanged (Figure 4 and Figure S4). The apparent disappearance of P_2_ indicated that P_0_ and P_1_ represented pre-chemistry conformational changes while P_2_ represented either the chemistry step or a post-chemistry event.

**Figure 5:**
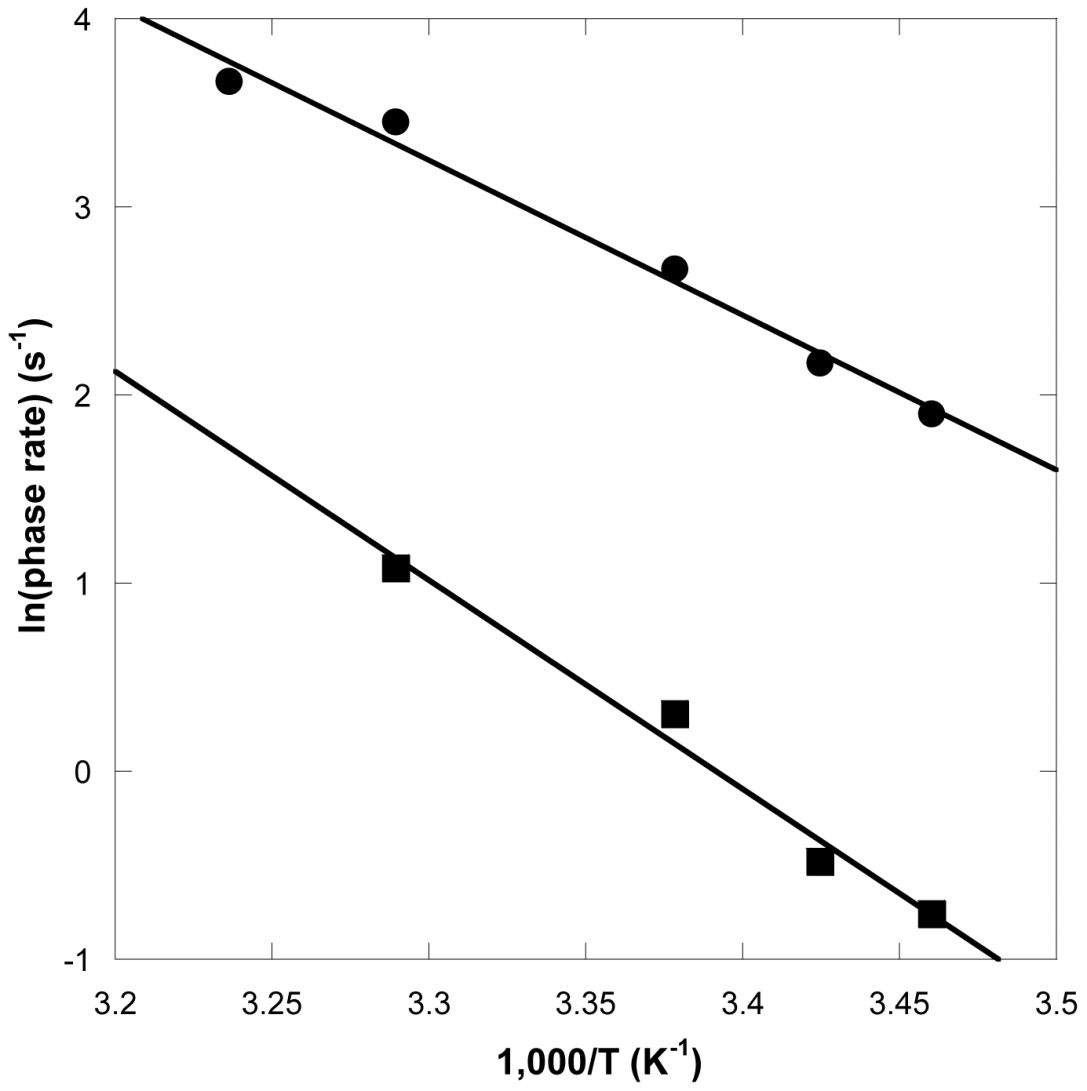
Activation energy barrier for dTTP incorporation into S-1 DNA catalyzed by the S112C Dpo4 mutant. The extracted rates of the P_1_ (circle) and P_2_ (square) phases were plotted as a function of reaction temperature to yield the activation energy barriers of 16.4±0.6 and 22±1 kcal/mol for P_1_ and P_2_, respectively.

Based on the varying temperature dependencies for the P_1_ and P_2_ rates at 20°C and 37°C, these data suggested that the free energy profile was different for these pre- and post-chemistry conformational transitions. Thus, parallel stopped-flow experiments were performed at 17°C, 24°C, and 32°C in order to determine the activation energy (*E*_a_) barriers for P_1_ and P_2_ (Table 2 and Figure S5). The rates for each phase were plotted as a function of temperature so that the *E*_a_ value could be extrapolated (Figure 5). Although the domain movements occurred at similar rates, the activation energy barriers showed a wider range: 13–18 kcal/mol for P_1_ and 20–25.4 kcal/mol for P_2_ (Table 2). The average *E*_a_ values were 15±2 and 23±2 kcal/mol for P_1_ and P_2_, respectively. Both of these *E*_a_ barriers were less than the *E*_a_ value of 32.9 kcal/mol, which has been determined previously as the rate-limiting conformational change prior to phosphodiester bond formation using a radioactive chemical-quench technique [20]. Therefore, neither of these fluorescent phases was directly related to the rate-limiting conformational change. Moreover, the *E*_a_ barrier for uncatalyzed phosphodiester bond formation in solution is estimated to be 21.1 kcal/mol [28]. The *E*_a_ should be lower than 21.1 kcal/mol if this reaction was catalyzed by an enzyme like Dpo4 based on Pauling's transition state theory [29]. Consistently, Florián et al. have used computer simulation to conclude that for T7 DNA polymerase, a rate-limiting phosphodiester bond formation step involving the transfer of a proton to activate the 3′-hydroxyl nucleophile accounts for an activation energy of 12.3 kcal/mol [28]. Radhakrishnan and Schlick have used quantum mechanics/molecular mechanics dynamics simulations and quasi-harmonic free energy calculations to show that the rate-limiting phosphodiester bond formation step for correct nucleotide incorporation catalyzed by DNA polymerase β occurs with a free energy of activation of 17 kcal/mol [30]. Thus, P_2_ likely represented a post-chemistry event rather than the chemistry step, because the average *E*_a_ of P_2_ was higher than that of uncatalyzed phosphodiester bond formation in solution. Also noteworthy, we focused on the FRET data collected at 20°C and 37°C, both sub-optimal temperatures for the thermostable Dpo4, since data collected at temperatures exceeding 37°C did not capture as many FRET phases due to the faster rates. Nonetheless, Dpo4 remained active, dynamic, and flexible at both 20°C and 37°C [20].

**Table 2:** The activation energy (*E*_a_) barriers for the P_1_ and P_2_ phases of selected Dpo4 mutants that monitored the domain motions relative to DNA.

Domain	Mutant^a^	*E*_a_ (kcal/mol)	
		P_1_	P_2_
Finger	N70C	17.4±0.2	24±1
	E49C	13.7±0.9	21.7±0.4
LF	R267C	15.6±0.8	24±1
	K329C	13±2	25.4±0.9
Thumb	K172C	15±2	20±2
	S207C	18±2	21±1
Palm	S112C	16.4±0.6	22±1
	N130C	17.8±0.7	24±2
Average		15±2	23±2

a Each of the Alexa594-labeled mutants contains the C31S mutation and is listed in Table S1.

### Motions of the Finger Domain Relative to the LF Domain during Catalysis

**Figure 6:**
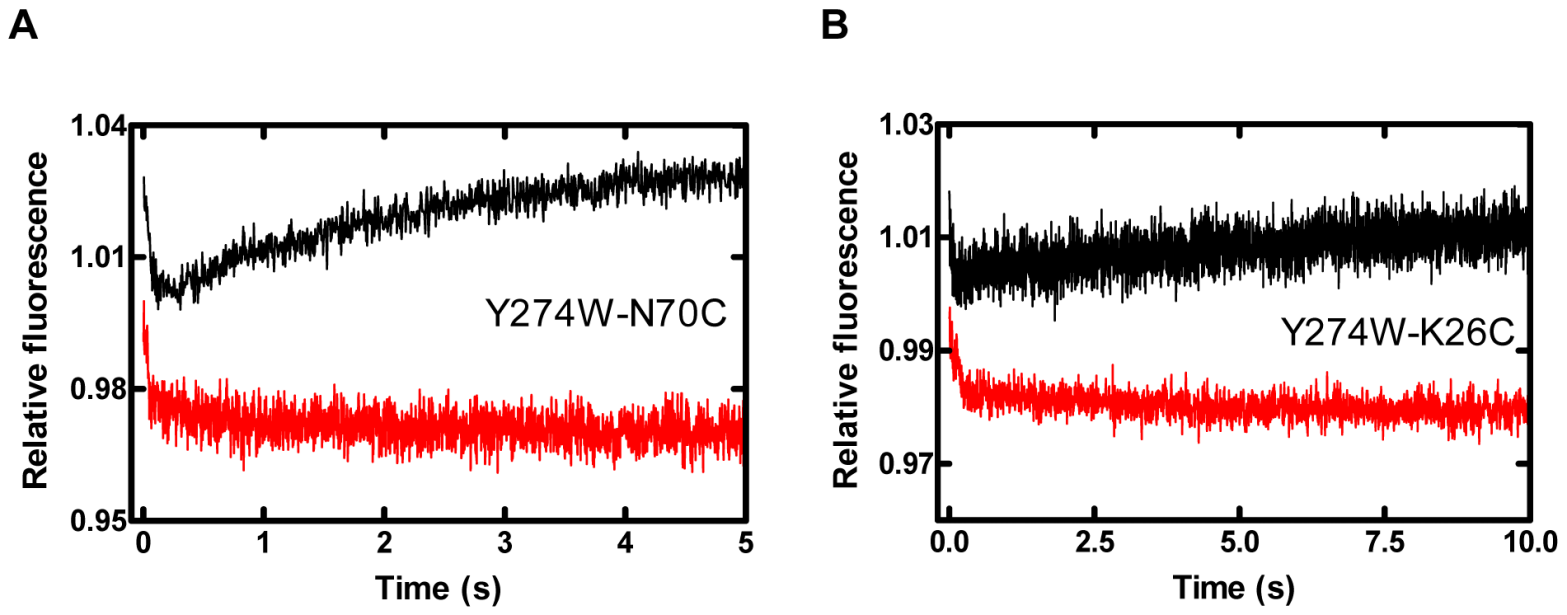
Stopped-flow kinetics of domain-domain motions at 20°C. A pre-incubated mixture of Dpo4 finger mutants (A) Y274W-N70C^CPM^ or (B) Y274W-K26C^CPM^ (200 nM) with either S-3 (black trace) or S-4 (red trace) DNA substrates (300 nM) was reacted with dTTP (1 mM). Only the fluorescence signals of acceptor CPM were recorded. Notably, some changes in fluorescence upon dTTP binding occurred during the instrument's dead time and the acceptor fluorescence signals at time zero or close to time zero were not recorded.

To support and expand upon the above work, the motions of the finger domain (Table S2) relative to the LF domain (Y274W) were investigated. For the two CPM-labeled Dpo4 mutants with S-3 (Table 1), the nucleotide binding and incorporation steps produced an acceptor fluorescence trace consisting of two phases: an initial, fast decrease phase followed by a slow increase phase (Figure 6, black trace). The similar kinetic rates, obtained after fitting each phase with a single-exponential equation, suggested that these two phases were correlated to P_1_ and P_2_ as identified from the above domain-DNA studies. Using dideoxy-terminated S-4 DNA, the second, slow phase was not detected (Figure 6, red trace). Together, these results demonstrated that the finger domain initially moved away from the LF domain before catalysis and then reopened following nucleotidyl transfer (Figure S6). Currently, we are investigating how the palm and thumb domains move relative the LF domain during correct nucleotide incorporation by using the real-time FRET methodology with the tryptophan/CPM as the FRET pair.

### Functional Implication of the Motion of the LF Domain

The overall picture emerging from our data suggested that the conserved polymerase core, composed of the finger, palm, and thumb domains, moved inward to tighten its grip on the DNA (Figures 1 and 3), which was important in aligning the substrates for formation of an active ternary complex (P_1_). In the meantime, the LF domain, a non-polymerase core domain, moved away from the DNA (Figures 1 and 3). After nucleotide incorporation, the domains slowly returned to a relaxed conformation (P_2_). The opposing directional movement of the LF domain may play a role in translesion synthesis. Functionally important domain rearrangements have been observed in many proteins [31]. By moving away from the DNA, the additional space at the polymerase active site may accommodate a distorted DNA structure, especially those containing bulky DNA lesions. Since this movement was observed with undamaged DNA, it is possible that the dynamic conformational motions of the LF domain have evolved to confer the lesion bypass abilities unique to Dpo4 and other Y-family DNA polymerases. Furthermore, the interactions between Dpo4 and the proliferating cell nuclear antigen have been mapped to the LF domain [32]. Therefore, this domain motion may be important during protein-protein interactions at the replication fork. Lastly, the inward movement of the LF domain during the post-chemistry relaxation (reopening) stage may inhibit the translocation of DNA and prevent processive nucleotide incorporation. This hypothesis is supported by the low polymerization processivity of Dpo4, which has been shown to be about one nucleotide incorporation per DNA binding event by us (K. A. Fiala and Z. Suo, unpublished data) and others [33].

### Rate-Limiting Step of Correct Nucleotide Incorporation

**Figure 7:**
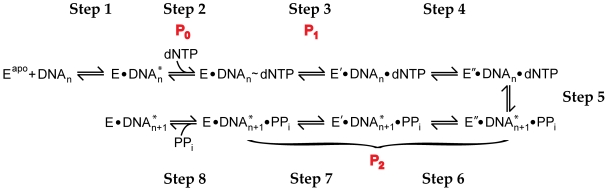
Mechanism of a single, correct nucleotide incorporation catalyzed by Dpo4. P_0_, P_1_, and P_2_ are the three phases observed in Figure 3. DNA* and DNA respectively represent the location of DNA in the active site of Dpo4 before and after DNA sliding by one base pair. E^apo^, E, E′, and E″ represent four different conformations of Dpo4. PP_i_ denotes pyrophosphate.

On the basis of our data, the minimal kinetic pathway catalyzed by Dpo4 [19] has been expanded as shown in Figure 7. In Step 1, the Dpo4•DNA binary complex was formed and existed mainly as a complex where the primer terminus occupied the dNTP binding pocket (DNA^*^) so that nucleotide incorporation could not occur until DNA translocated [8]. Notably, the structure of Dpo4 undergoes significant conformational changes from its apo form (E^apo^) to its binary form based on our published structural studies [8]. Step 2, which corresponded to the FRET signal change of P_0_, demonstrated the DNA translocation event induced by nucleotide binding [8],[9]. Once the ternary complex has formed, Dpo4 tightened its grip (E′) as evident by the domain motions representing P_1_ (Step 3). Superimposing the crystal structures of Dpo4's binary and ternary complexes has revealed that some active site residues are re-positioned (E″) to properly align all substrates (Step 4), and this process corresponded to the rate-limiting step during nucleotide incorporation as determined by our previous work (see discussion below) [8],[19],[20]. Following phosphodiester bond formation (Step 5), the active site isomerisation step must be reversed (Step 6) as well as the “grip” conformational change (Step 7), i.e., the conformational transition related to the fluorescence change observed in P_2_. Lastly, the reopening of the domains allowed pyrophosphate (PP_i_) to be released (Step 8) so that the binary complex can either undergo another catalytic cycle or dissociate [19],[20].

The assignment of the rate-limiting step during nucleotide incorporation has been controversial in the DNA polymerase field for a long time [21]. We propose that Step 4 represents the rate-limiting event (Figure 7) for the following reasons: (i) at 20°C, the collapse of the polymerase core domains (Step 3) was much faster (average P_1_ = 15.3 s^−1^ in Table S4) than the rates determined using radioactive chemical-quench techniques (average *k_obs_* = 0.66 s^−1^), which is consistent with the fast closure rate of the finger domain of other DNA polymerases [16]–[18]; (ii) the rate of phosphodiester bond formation is estimated to be 9,000 s^−1^ at 20°C [34]; and (iii) the activation energy barriers of the P_1_ and P_2_ conformational transitions did not coincide with the *E*_a_ of 32.9 kcal/mol obtained for nucleotide incorporation (see above discussion) [20]. The evidence in (i) and (iii) exclude Step 3 as rate limiting while both (ii) and (iii) eliminated Step 5 in our consideration. These differences in rate and *E*_a_ barriers along with three independent lines of kinetic evidence (about 25% more products can be formed if the reaction is chased with a large excess of unlabeled, correct dTTP, rather than quenched with strong acid; E″•DNA_n_•dNTP has a ∼100-fold slower dissociation rate than E•DNA_n_•dNTP; and there is an insignificant elemental effect between the incorporation of correct dTTP and its α-thio analog, Sp-dTTPαS [19],[20]) suggested that the pre-chemistry isomerisation step (Step 4) limited the rate of a correct nucleotide incorporation determined using radioactive chemical-quench techniques. Thus, Step 4 occurred at an average rate of 0.66 s^−1^ at 20°C. However, this rate-limiting step was not probed here because the subtle active site rearrangements would not alter the distance between the FRET pair, thereby yielding no detectable FRET signal changes. Currently, the nature of Step 4 is unclear. It may involve repositioning of the side chains of active site residues [8], binding of metal ion(s) [12], and/or realignment of the 3′-hydroxyl of the primer terminus and the α-phosphate of an incoming nucleotide for an in-line phosphodiester bond formation [35]. Notably, the rates of P_2_ at 20°C (average P_2_ = 0.57 s^−1^) were similar to the rapid-chemical quench rates (average *k_obs_* = 0.66 s^−1^). This is because the P_2_ fluorescence signal likely originated from the rapid domain movements that occurred during Step 7. However, the rate was limited by the slow, preceding isomerisation process (Step 6). Although we do not know the rate of Step 6, we assume that it was comparable to the rate of Step 4, since Step 6 was the reverse isomerisation process.

### Magnitudes of Protein Conformational Changes during Catalysis

To determine the magnitudes of the protein conformational changes in Figure 7, we quantitatively estimated the distances between the donor and acceptor fluorophores in FRET system (i) (see above) using the measurements of steady-state FRET efficiency at 20°C as in Figure 2 (unpublished data). Distances were calculated for each of the two states (Table S5): the initial binary complex of Dpo4 and S-1 (i.e., E•DNA_n_* in Figure 7) and the ternary complex of Dpo4, S-2, and dTTP (i.e., E″•DNA_n_•dNTP in Figure 7). Accordingly, the net movements for nine of Dpo4's residues during Steps 2 through 4 in Figure 7 vary from residue to residue and were in the range of −0.02 to 1.52 Å (Table S5, positive values indicate that the Dpo4 residues moved away from DNA). Consistently, if the residue moved away from the DNA during Step 3 as suggested by the above real-time FRET during P_1_, e.g., E49, K329, and R267, then the net movement value in Table S5 is positive and relatively large since both the DNA translocation event in Step 2 and the conformational change in Step 3 increased the distance between the FRET pair. Interestingly, these steady-state FRET efficiency-based values were close to the predicted net movements (−0.59 to 3.95 Å) of the corresponding Dpo4 residues during correct nucleotide binding based on the binary [8] and ternary [36] crystal structures of Dpo4 (Table S6). If DNA slides by one base pair in Step 2 as suggested by the crystal structures of Dpo4 [8], then the changes in distance between the nine FRET pairs due to movement of the DNA were predicted to be in the range of −1.35 to 5.21 Å (Table S6). Moreover, the motion distances of these nine Dpo4 residues from Steps 3 to 4, which were likely dominated by Step 3, were predicted to be in the range of either −3.69 to 1.86 Å (Table S5) or −1.51 to 0.93 Å (Table S6). Together, these measured and predicted data suggest that the motions of Dpo4's residues and domains as induced by the binding of a correct dTTP were not dramatic and occurred within a few angstroms.

Although trends of residue motions derived from Tables S5 and S6 were similar, the structurally predicted net movements during Steps 2 to 4 are larger. These differences are not surprising since the crystal structures that are often influenced by crystal packing may not reflect the exact structures in solution. Furthermore, the flexibility of the long linker for fluorophore attachment to a cysteine residue or a DNA base may induce uncertainty in the estimated distances between FRET pairs based on the steady-state FRET efficiencies. Lastly, distance calculations in Table S5 assumed that both the donor and acceptor fluorophores can undergo unrestricted isotropic motions, which may not be true for the various conformations sampled by each measured residue in Dpo4.

### Conclusions and Future Directions

The combined dynamic and kinetic studies allow us to draw four conclusions that improve our current understanding about the kinetic mechanism of DNA synthesis (Figure 7). First, there was a rapid DNA translocation event induced by the binding of a correct nucleotide. Second, the four domains of Dpo4 moved in a synchronized manner during correct nucleotide incorporation. The LF domain and the polymerase core moved in opposite directions. The palm and finger domains did not move as rigid bodies due to the presence of intra-domain rotational movements. Third, the motions of the amino acid residues and domains of Dpo4 induced by correct nucleotide binding are within a few angstroms. Fourth, the active site rearrangement process (Step 4), rather than the pre-chemistry conformational change associated with domain movements (Step 3) and phosphodiester bond formation (Step 5), limited the rate of correct nucleotide incorporation in the reaction pathway. Moreover, Step 3 (15 kcal/mol), Step 4 (32.9 kcal/mol), Step 5 (<21.1 kcal/mol), and Step 6 (23 kcal/mol) were thermodynamically distinguished in this paper.

In addition to the invaluable information gathered on the protein dynamics of Dpo4, our study illustrated the limitations of monitoring the motions of only a single residue relative to DNA by stopped-flow FRET [16]–[18] as seen by the contrasting results observed for residues on the finger and palm domains of Dpo4 in this paper. By monitoring multiple residues, we were able to reveal the proposed rotational nature of the domain movements. It is possible that further rotations or semi-rigid domain motions could be determined by monitoring more sites on the protein or DNA. These measurements are more meaningful if amino acid residues in each domain of Dpo4 move with slightly different rates and/or directions in each of the protein conformational change steps (Step 3, Step 4, Step 6, and Step 7) as the slightly different P_1_ and P_2_ rates in Table S4 have suggested (Figure 7). Thus, by using the real-time FRET methodology, this study presents a powerful system for monitoring the global dynamics of protein motions at multiple sites, which is necessary to gain a better understanding of enzyme catalysis.

At present, we are using this system to investigate protein dynamics during incorrect nucleotide incorporation. It will be interesting to see whether DNA also translocates in order to free space for the binding of an incorrect nucleotide, whether Dpo4 undergoes similar global conformational dynamics as described above, whether Dpo4 uses a similar kinetic mechanism as shown in Figure 7, and whether Step 4 is rate-limiting during misincorporation. Differences in the kinetic mechanisms for correct and incorrect nucleotide incorporations will reveal which steps serve as kinetic checkpoints and help Dpo4 to achieve its fidelity [21]. Moreover, since Dpo4 functions as a lesion bypass DNA polymerase in vivo, we are employing our FRET systems to explore Dpo4's protein dynamics during the bypass of DNA lesions, including an abasic site [37], a *N*-(deoxyguanosin-8-yl)-1-aminopyrene adduct [26], and a cisplatin-DNA adduct [38]. These studies will reveal how a lesion in the DNA template affects concerted domain motions within Dpo4 during DNA synthesis.

## Materials and Methods

### Preparation of Dpo4 Mutants and DNA Substrates

The plasmid [39] encoding the *dpo4* gene from *S. solfataricus* P2 was mutated using the Stratagene QuikChange kit. To avoid ambiguity of labeling, the sole native cysteine was replaced with a serine (C31S). Using the C31S mutant as a template, single cysteine substitutions were introduced individually into each domain (Table S1). Separately, an endogenous tryptophan FRET donor was substituted into the LF domain by generating a Y274W mutant (Table S2). All mutants summarized in Tables S1 and S2 contain the C31S substitution. Mutations were confirmed by DNA sequencing (OSU Plant-Microbe Genomics Facility). Purification of the mutant proteins was performed as described for wild-type Dpo4 [39].

Dpo4 mutants were labeled with either Alexa594 or CPM (Molecular Probes, Invitrogen) by incubating the mixture at 4°C for 12 h with a 10-fold molar excess of dye, according to the manufacturer's protocol. After labeling, each Dpo4 mutant was separated from the unbound fraction of dye by both size-exclusion chromatography (G-25 resin) and extensive dialysis. The labeling efficiencies were typically 95% or greater as determined by the Bradford protein assay (Bio-Rad). The protein concentration of each dye-labeled Dpo4 mutant was determined by a spectrometric Bradford protein assay (Bio-Rad) by using the corresponding unlabeled Dpo4 mutant as a protein standard. The concentration of each unlabeled protein was determined by UV spectroscopy at 280 nm using the calculated molar extinction coefficient of 28,068 M^−1^ cm^−1^.

All oligonucleotides (Table 1) were purchased from Integrated DNA Technologies. Alexa488 (Molecular Probes, Invitrogen) was attached to a 5-C6-Amino-2′-deoxythymidine on the ninth primer base from the 3′-end of the DNA substrates. Alexa488-labeled DNA was purified according to the manufacturer's protocol and annealed as described previously [39].

### Kinetics Experiments

Steady-state fluorescent assays (Fluoromax-3, Jobin Yvon Horiba), stopped-flow kinetic assays (Applied Photophysics SX20, UK), and rapid chemical-quench kinetic assays (KinTek) were carried out under the same conditions in buffer R, which contained 50 mM HEPES, pH 7.5 at desired temperature, 50 mM NaCl, 6 mM MgCl_2_, 0.1 mM EDTA, and 10% glycerol. For domain motions relative to DNA, 600 nM Dpo4 mutant, 100 nM DNA, and 1 mM dTTP were used. For stopped-flow experiments, with excitation of donor Alexa488 at 493 nm, both donor and acceptor fluorescence signals were recorded separately by using band pass filters XF3084 for Alexa488 (band pass range: 510–570 nm, Omega Optical, USA) and XF3028 for Alexa594 (band pass range: 615–650 nm, Omega Optical, USA) over time. For finger domain motions relative to the LF, 200 nM Dpo4 mutant, 300 nM DNA, and 1 mM dTTP were reacted. The CPM fluorescence was monitored by using a 420-nm cut-off filter when the tryptophan donor was excited at 290 nm. In both steady-state and stopped-flow kinetic experiments, slits were set at 5 nm for both excitation and emission. Fluorescence traces were fit to a single-exponential equation, ΔF = A[exp(−*k*t)]+constant. Rapid chemical-quench reactions [39] were performed as described previously. For each reaction time course, a single-exponential equation, [Product] = A[1−exp(−*k_obs_*t)], was used to extract the observed rate constant (*k_obs_*). Activation energy barriers were extrapolated as described previously [20]. Briefly, the plot of ln*k* versus 1/T was fit to a linear equation, ln*k* = −*E*_a_/RT + constant, to extract the activation energy barrier (*E*_a_). “*k*” was the rate derived from the stopped-flow experiment at each reaction temperature T (Kelvin).

## Supporting Information

Figure S1 **Circular dichroism spectra of wild-type Dpo4 and Dpo4 mutants at 37°C.** Circular dichroism spectra were collected on Model 62A DS Spectrometer (Aviv, Lakewood, NJ) in a 1-mm path-length cuvette at 37°C. The spectra were taken in the buffer (25 mM sodium phosphate, pH 7.5, 50 mM NaCl, and 5 mM MgCl_2_). Data points were recorded from 270 to 200 nm at 1-nm intervals. Each data point was averaged for 5 s. Wild-type Dpo4 (40 kDa) is shown in black while double-point mutants (Table S1) are shown in color (N70C in purple, S112C in red, S207C in green, and K329C in blue).
(0.10 MB TIF)

Figure S2 **Control experiments of finger domain mutant (N70C) by steady-state and stopped-flow FRET under the same reaction conditions at 20°C.** The reaction condition: 600 nM protein, 100 nM DNA, and 1 mM dTTP. The mixture was excited at a wavelength of 493 nm. Steady-state control experiments of (A) the unlabeled protein and labeled DNA and (B) the labeled protein and unlabeled DNA. The black trace shows the overall fluorescence of DNA alone. Addition of protein and dTTP (1 mM) produced the red and green traces, respectively. Notably, the red and green traces in (A) are superimposible. The red-dashed trace was extracted from Figure 2A and is shown here for comparison with the non-FRET (background) acceptor signal. Spectra were normalized to 1 by using the donor as a reference. (C) Stopped-flow control experiments were performed with either unlabeled protein and labeled DNA (green trace) or labeled protein and unlabeled DNA (red trace) in the presence of dTTP (1 mM) as described in Figure 3.
(0.24 MB TIF)

Figure S3 **Stopped-flow kinetics of dTTP incorporation into a normal DNA substrate S-1 at 20°C.** Dpo4 mutant•S-1 DNA complexes were reacted with dTTP and the fluorescence of the donor (green) and acceptor (red) was recorded individually. The traces are shown for (A) the finger (E49C), (B) palm (S96C), (C) palm (N130C), (D) LF (R267C), and (E) thumb (K172C). Each of these mutants also contained the C31S mutation and was labeled with Alexa594 (Table S1). DNA substrate S-1 was labeled with Alexa488. Notably, some changes in fluorescence upon dTTP binding occurred during the instrument's dead time and the donor and acceptor fluorescence signals at time zero or close to time zero were not recorded.
(0.31 MB TIF)

Figure S4 **Stopped-flow kinetics of dTTP incorporation into a dideoxy-terminated DNA substrate S-2 at 20°C.** Dpo4 mutant•S-2 DNA complexes were reacted with dTTP and the fluorescence was monitored using a stopped-flow apparatus. Donor (green) and acceptor (red) traces are shown for the (A) finger (E49C), (B) palm (S96C), (C) palm (N130C), (D) LF (R267C), and (E) thumb (K172C) domains. Each of these mutants also contained the C31S mutation and was labeled with Alexa594 (Table S1). DNA substrate S-2 was labeled with Alexa488. Notably, some changes in fluorescence upon dTTP binding occurred during the instrument's dead time and the donor and acceptor fluorescence signals at time zero or close to time zero were not recorded.
(0.39 MB TIF)

Figure S5 **Stopped-flow kinetics of dTTP incorporation into a normal DNA substrate S-1 catalyzed by a Dpo4 mutant (S112C) at different temperatures.** Dpo4 mutant (S112C)•S-1 DNA complexes were reacted with dTTP. The stopped-flow experiments were performed at (A) 17°C, (B) 20°C, (C) 24°C, (D) 32°C, and (E) 37°C. The traces monitoring donor and acceptor fluorescence are shown in green and red, respectively. The Dpo4 mutant also contained the C31S mutation and was labeled with Alexa594 (Table S1). DNA substrate S-1 was labeled with Alexa488. Notably, some changes in fluorescence upon dTTP binding occurred during the instrument's dead time and the donor and acceptor fluorescence signals at time zero or close to time zero were not recorded.
(0.36 MB TIF)

Figure S6 **Finger domain motions relative to the LF domain during a single, correct nucleotide incorporation.** The domains of Dpo4 are shown in blue (finger), red (palm), green (thumb), and purple (LF); the DNA is in gold; acceptor CPM-labeled mutant residues are in yellow and the single mutant tryptophan donor is in cyan. The arrows (black for CPM-labeled residues, white for Y274W) represent the direction of movement based on the FRET signals from both domains relative to DNA and relative to LF domain experiments for (A) phase P_1_ and (B) phase P_2_. Structures are shown in a different view from those in Figure 1.
(3.05 MB TIF)

Table S1 **Dpo4 mutants for monitoring the domain motions relative to DNA.**
(0.04 MB DOC)

Table S2 **Dpo4 mutants for monitoring the finger domain motions relative to the little finger domain.**
(0.03 MB DOC)

Table S3 **The rates of correct nucleotide incorporation catalyzed by unlabeled- and dye-labeled Dpo4 mutants under single-turnover conditions.** The radioactive experiments were performed in a rapid-chemical quench apparatus.
(0.05 MB DOC)

Table S4 **The phase rates (s^−1^) derived from stopped-flow kinetic assays are the average from multiple independent experiments and are reported as mean ± standard deviation.**
(0.08 MB DOC)

Table S5 **Measured motion distances of each Alexa594-labelled amino acid residue of Dpo4 during correct nucleotide binding at 20°C using steady-state FRET data.**
(0.05 MB DOC)

Table S6 **Predicted motion distances of each selected amino acid residue of Dpo4 during correct nucleotide binding on the basis of the binary and ternary crystal structures of Dpo4.**
(0.05 MB DOC)

## Abbreviations

CPM: 7-diethylamino-3-(4′-maleimidylphenyl)-4-methylcoumarin
Dbh: DinB homolog
Dpo4: *Sulfolobus solfataricus* DNA polymerase IV
FRET: fluorescence resonance energy transfer
LF: litter finger
