# Supplementary figures and images for: Global Conformational Dynamics of a Y-Family DNA Polymerase during Catalysis
Source: PLoS Biol. 2009 Oct 27;7(10):e1000225. doi: 10.1371/journal.pbio.1000225 (PMC2758995; doi:10.1371/journal.pbio.1000225)

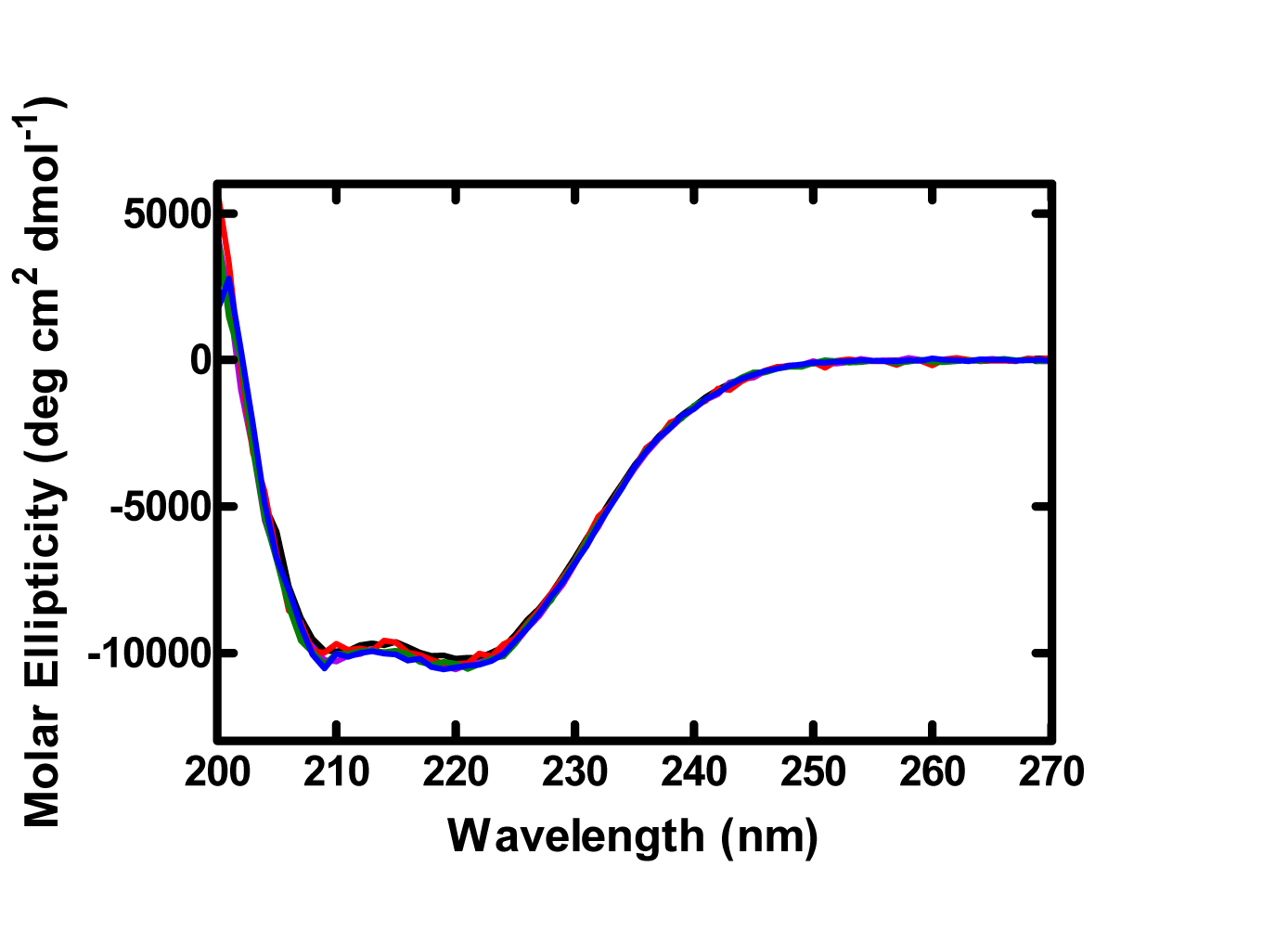

Supplement: Figure S1 — Circular dichroism spectra of wild-type Dpo4 and Dpo4 mutants at 37°C. Circular dichroism spectra were collected on Model 62A DS Spectrometer (Aviv, Lakewood, NJ) in a 1-mm path-length cuvette at 37°C. The spectra were taken in the buffer (25 mM sodium phosphate, pH 7.5, 50 mM NaCl, and 5 mM MgCl2). Data points were recorded from 270 to 200 nm at 1-nm intervals. Each data point was averaged for 5 s. Wild-type Dpo4 (40 kDa) is shown in black while double-point mutants (Table S1) are shown in color (N70C in purple, S112C in red, S207C in green, and K329C in blue). (0.10 MB TIF) [file pbio.1000225.s001.tif]

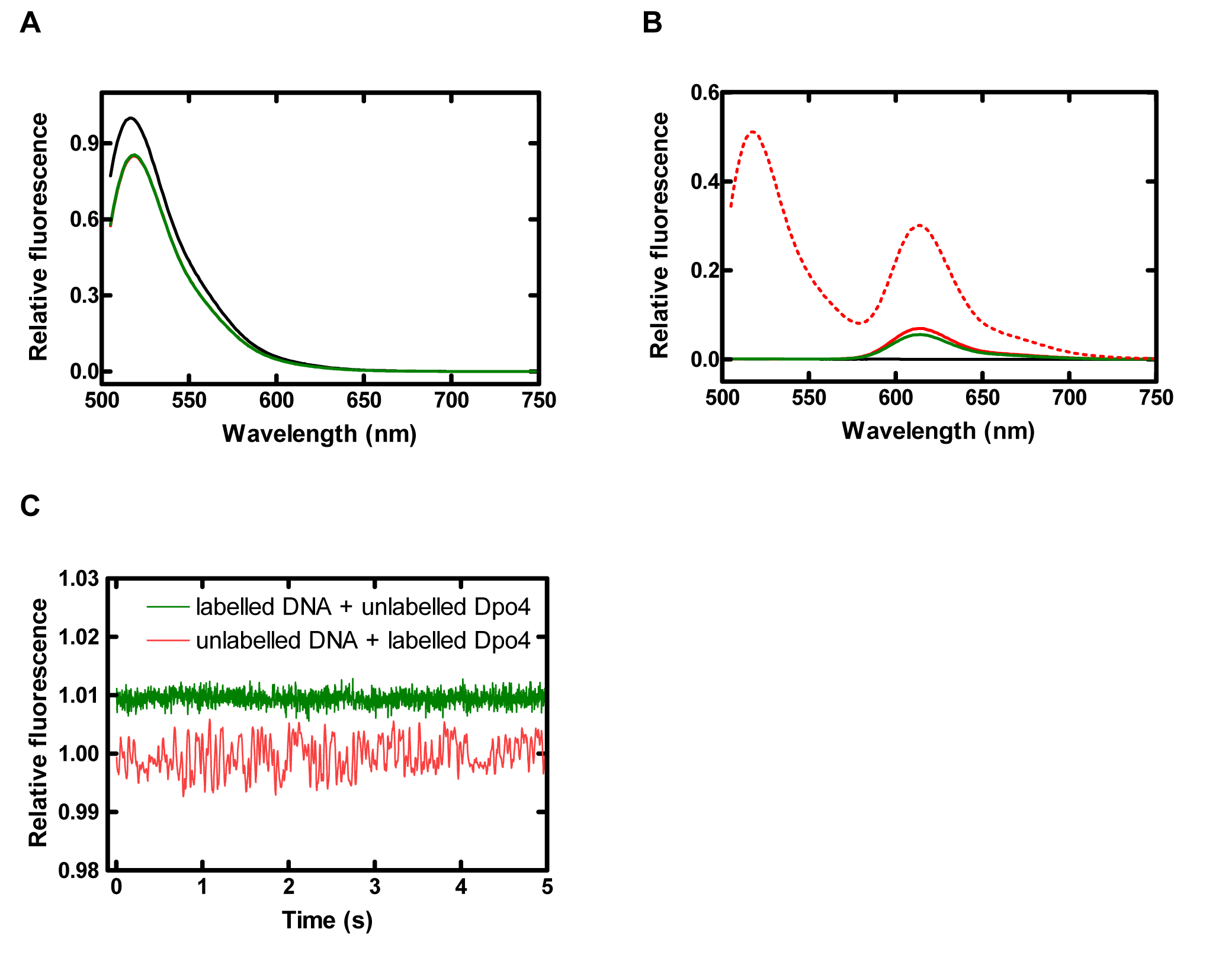

Supplement: Figure S2 — Control experiments of finger domain mutant (N70C) by steady-state and stopped-flow FRET under the same reaction conditions at 20°C. The reaction condition: 600 nM protein, 100 nM DNA, and 1 mM dTTP. The mixture was excited at a wavelength of 493 nm. Steady-state control experiments of (A) the unlabeled protein and labeled DNA and (B) the labeled protein and unlabeled DNA. The black trace shows the overall fluorescence of DNA alone. Addition of protein and dTTP (1 mM) produced the red and green traces, respectively. Notably, the red and green traces in (A) are superimposible. The red-dashed trace was extracted from Figure 2A and is shown here for comparison with the non-FRET (background) acceptor signal. Spectra were normalized to 1 by using the donor as a reference. (C) Stopped-flow control experiments were performed with either unlabeled protein and labeled DNA (green trace) or labeled protein and unlabeled DNA (red trace) in the presence of dTTP (1 mM) as described in Figure 3. (0.24 MB TIF) [file pbio.1000225.s002.tif]

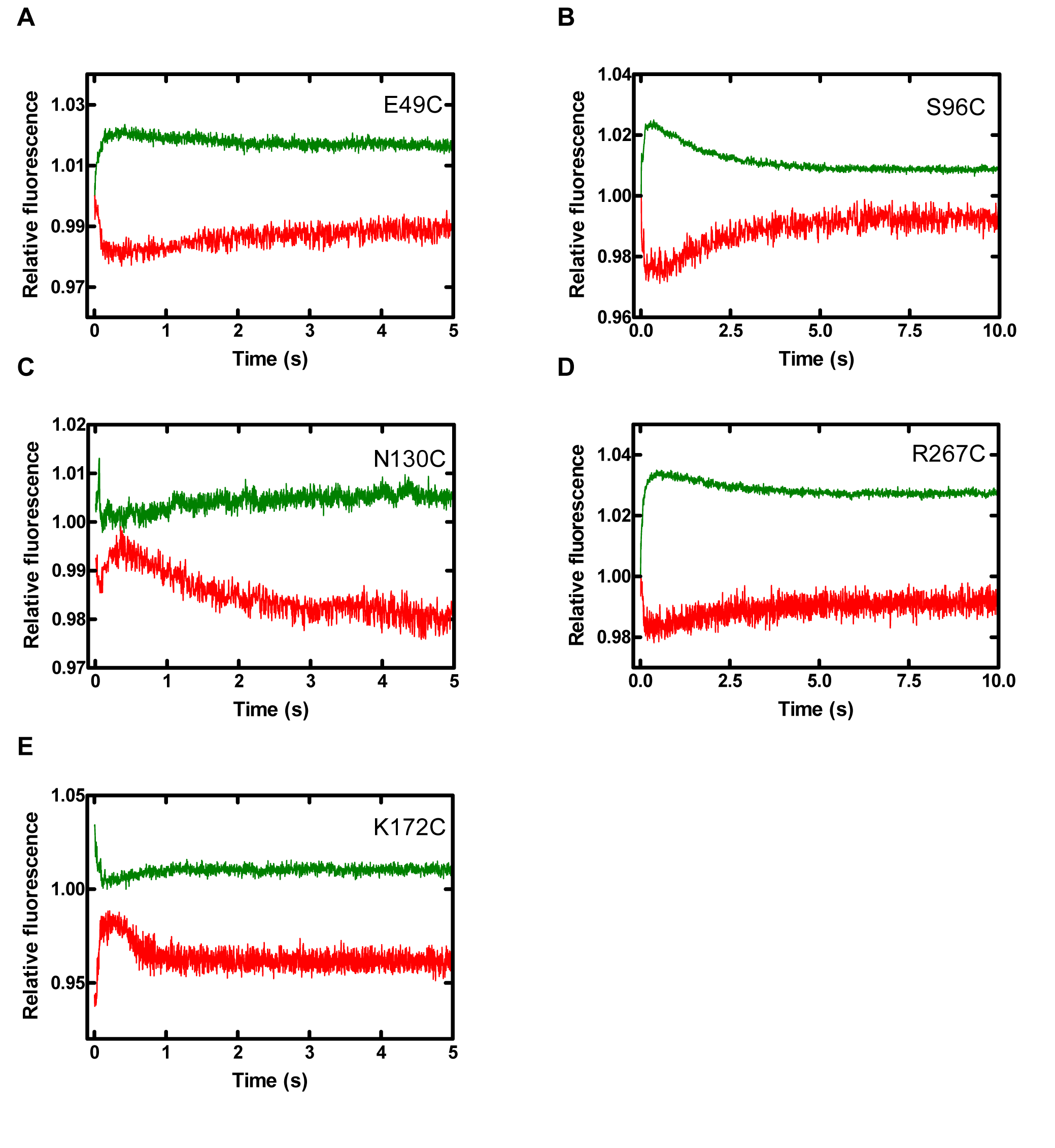

Supplement: Figure S3 — Stopped-flow kinetics of dTTP incorporation into a normal DNA substrate S-1 at 20°C. Dpo4 mutant•S-1 DNA complexes were reacted with dTTP and the fluorescence of the donor (green) and acceptor (red) was recorded individually. The traces are shown for (A) the finger (E49C), (B) palm (S96C), (C) palm (N130C), (D) LF (R267C), and (E) thumb (K172C). Each of these mutants also contained the C31S mutation and was labeled with Alexa594 (Table S1). DNA substrate S-1 was labeled with Alexa488. Notably, some changes in fluorescence upon dTTP binding occurred during the instrument's dead time and the donor and acceptor fluorescence signals at time zero or close to time zero were not recorded. (0.31 MB TIF) [file pbio.1000225.s003.tif]

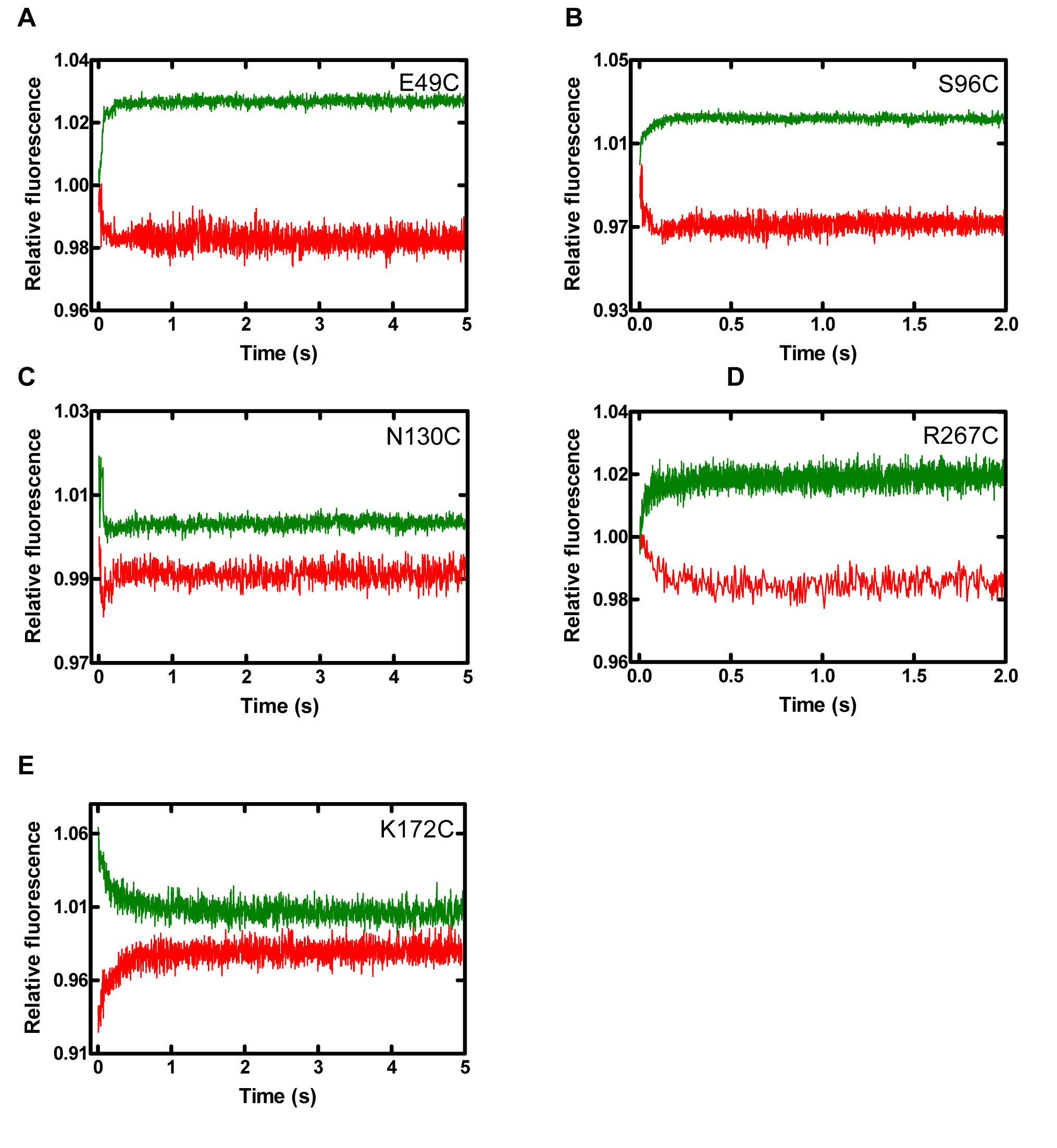

Supplement: Figure S4 — Stopped-flow kinetics of dTTP incorporation into a dideoxy-terminated DNA substrate S-2 at 20°C. Dpo4 mutant•S-2 DNA complexes were reacted with dTTP and the fluorescence was monitored using a stopped-flow apparatus. Donor (green) and acceptor (red) traces are shown for the (A) finger (E49C), (B) palm (S96C), (C) palm (N130C), (D) LF (R267C), and (E) thumb (K172C) domains. Each of these mutants also contained the C31S mutation and was labeled with Alexa594 (Table S1). DNA substrate S-2 was labeled with Alexa488. Notably, some changes in fluorescence upon dTTP binding occurred during the instrument's dead time and the donor and acceptor fluorescence signals at time zero or close to time zero were not recorded. (0.39 MB TIF) [file pbio.1000225.s004.tif]

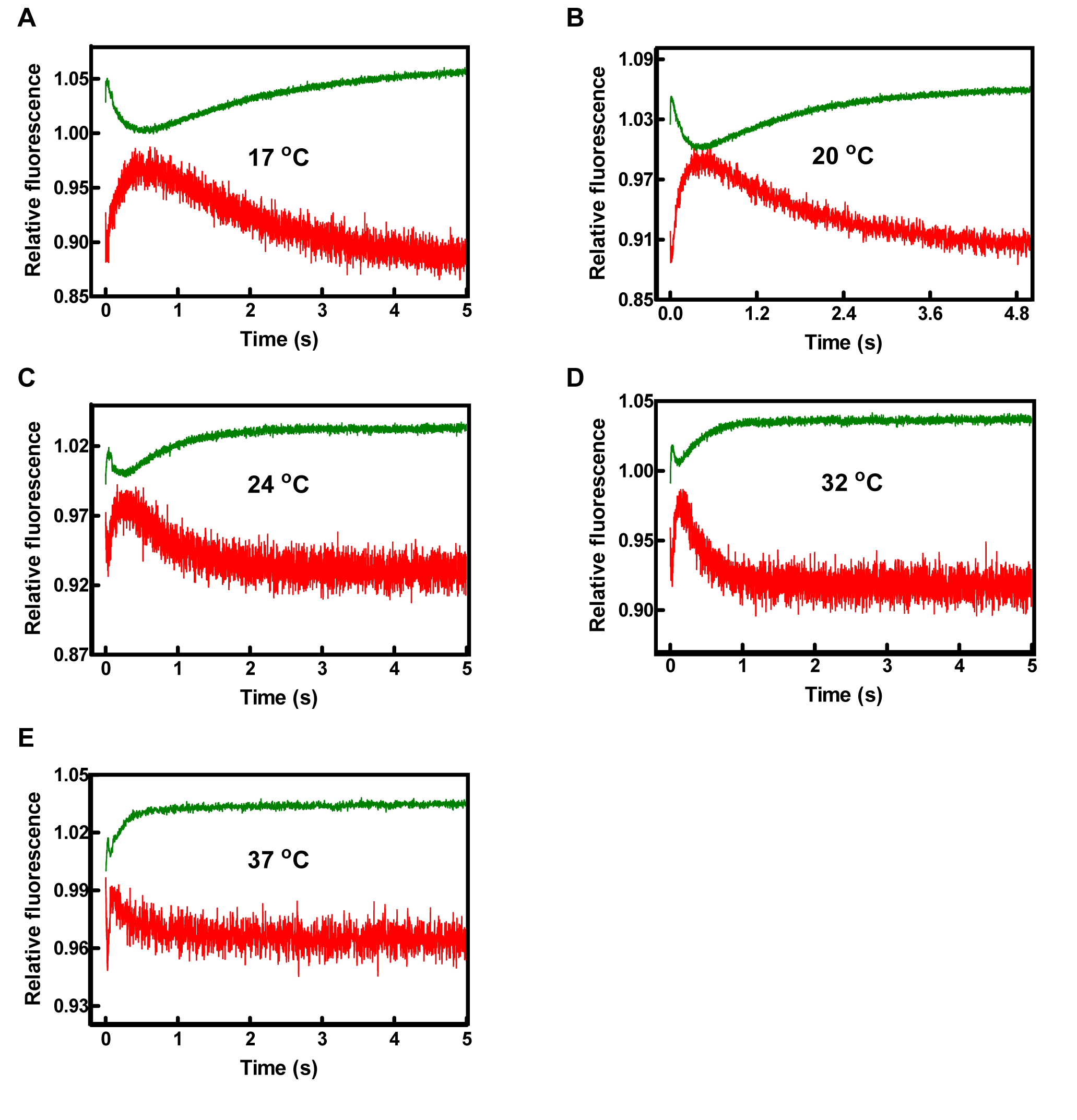

Supplement: Figure S5 — Stopped-flow kinetics of dTTP incorporation into a normal DNA substrate S-1 catalyzed by a Dpo4 mutant (S112C) at different temperatures. Dpo4 mutant (S112C)•S-1 DNA complexes were reacted with dTTP. The stopped-flow experiments were performed at (A) 17°C, (B) 20°C, (C) 24°C, (D) 32°C, and (E) 37°C. The traces monitoring donor and acceptor fluorescence are shown in green and red, respectively. The Dpo4 mutant also contained the C31S mutation and was labeled with Alexa594 (Table S1). DNA substrate S-1 was labeled with Alexa488. Notably, some changes in fluorescence upon dTTP binding occurred during the instrument's dead time and the donor and acceptor fluorescence signals at time zero or close to time zero were not recorded. (0.36 MB TIF) [file pbio.1000225.s005.tif]

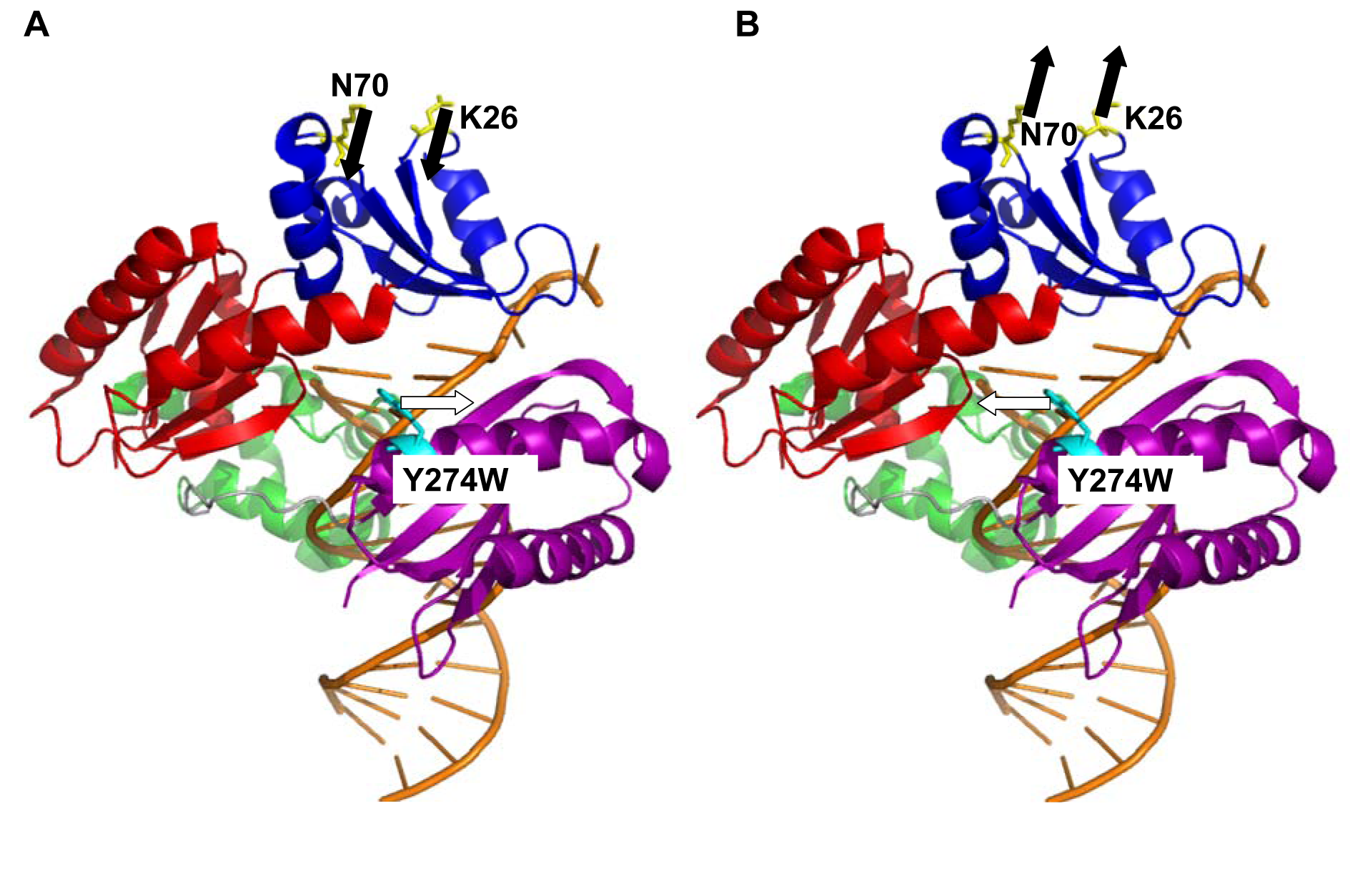

Supplement: Figure S6 — Finger domain motions relative to the LF domain during a single, correct nucleotide incorporation. The domains of Dpo4 are shown in blue (finger), red (palm), green (thumb), and purple (LF); the DNA is in gold; acceptor CPM-labeled mutant residues are in yellow and the single mutant tryptophan donor is in cyan. The arrows (black for CPM-labeled residues, white for Y274W) represent the direction of movement based on the FRET signals from both domains relative to DNA and relative to LF domain experiments for (A) phase P1 and (B) phase P2. Structures are shown in a different view from those in Figure 1. (3.05 MB TIF) [file pbio.1000225.s006.tif]
