# Supplementary material for: Global Conformational Dynamics of a Y-Family DNA Polymerase during Catalysis
Source: PLoS Biol. 2009 Oct 27;7(10):e1000225. doi: 10.1371/journal.pbio.1000225 (PMC2758995; doi:10.1371/journal.pbio.1000225)
Supplement: Table S1 — Dpo4 mutants for monitoring the domain motions relative to DNA. (0.04 MB DOC) [file pbio.1000225.s007.doc]

| **Table S1.** Dpo4 mutants for monitoring the domain motions relative to DNA. | | | |
| --- | --- | --- | --- |
| **Domain** | **Mutant**^a^ | **Distance**^b^ (Å) | **Location of Acceptor**^c^ |
| Finger | N70C^Alexa594^ | 54.0 | at the loop between α-helix C and β-sheet 4 |
|  | E49C^Alexa594^ | 49.2 | within α-helix B |
| Palm | S96C^Alexa594^ | 36.2 | at the loop between α-helix D and β-sheet 5 |
|  | S112C^Alexa594^ | 36.7 | at the loop between β-sheet 6 and a 3_10_-helix |
|  | N130C^Alexa594^ | 47.7 | within α-helix E |
| Thumb | S207C^Alexa594^ | 34.7 | at the loop between α-helices I and J |
|  | K172C^Alexa594^ | 39.3 | within α-helix G |
| Little Finger | K329C^Alexa594^ | 32.4 | at the loop between α-helix M and β-sheet 12 |
|  | R267C^Alexa594^ | 32.5 | within α-helix L |
| ^a^All mutants contain the C31S mutation.  ^b^Estimated distance between the donor Alexa488 on DNA and acceptor Alexa594 on Dpo4’s domains based on the ternary crystal structure in [22]. Each distance is within R_0_ ± 0.5R_0_ (R_0_ = 60 Å) for efficient FRET.  ^c^Structural motifs are named as in [22]. | | | |
