# Supplementary material for: Global Conformational Dynamics of a Y-Family DNA Polymerase during Catalysis
Source: PLoS Biol. 2009 Oct 27;7(10):e1000225. doi: 10.1371/journal.pbio.1000225 (PMC2758995; doi:10.1371/journal.pbio.1000225)
Supplement: Table S2 — Dpo4 mutants for monitoring the finger domain motions relative to the little finger domain. (0.03 MB DOC) [file pbio.1000225.s008.doc]

| **Table S2.** Dpo4 mutants for monitoring the finger domain motions relative to the little finger domain. | | |
| --- | --- | --- |
| **Mutant**^a^ | **Distance**^b^ (Å) | **Location of Acceptor**^c^ |
| Y274W-N70C^CPM^ | 36.6 | at the loop between α-helix C and β-sheet 4 |
| Y274W-K26C^CPM^ | 35.4 | at the loop between a 3_10_-helix and β-sheet 2 |
| ^a^All mutants contain the C31S mutation.  ^b^Estimated distance between the donor Y274W on the little finger domain and CPM acceptor on the finger domain of Dpo4 based on the ternary crystal structure in [22]. CPM represents 7-diethylamino-3-(4′-maleimidylphenyl)-4-methylcoumarin. Each distance is within R_0_ ± 0.5R_0_ (R_0_ = 30 Å) for efficient FRET.  ^c^Structural motifs are denoted as in [22]. | | |
