# Supplementary material for: Global Conformational Dynamics of a Y-Family DNA Polymerase during Catalysis
Source: PLoS Biol. 2009 Oct 27;7(10):e1000225. doi: 10.1371/journal.pbio.1000225 (PMC2758995; doi:10.1371/journal.pbio.1000225)
Supplement: Table S3 — The rates of correct nucleotide incorporation catalyzed by unlabeled- and dye-labeled Dpo4 mutants under single-turnover conditions. The radioactive experiments were performed in a rapid-chemical quench apparatus. (0.05 MB DOC) [file pbio.1000225.s009.doc]

| **Table S3.** The rates of correct nucleotide incorporation catalyzed by unlabelled- and dye-labelled Dpo4 mutants under single-turnover conditions. The radioactive experiments were performed in a rapid-chemical quench apparatus. | | | |
| --- | --- | --- | --- |
| **Domain** | **Un-/labelled Protein** | ***k_obs_***^a^ (s^-1^) | |
|  |  | **20 °C** | **37 °C** |
|  | WT Dpo4 | 0.8±0.1 | 9±2 |
| Finger | N70C | 0.7±0.1 | 7.4±0.8 |
|  | E49C | 0.8±0.1 | 7.8±0.9 |
|  | N70C^Alexa594^ | 0.60±0.04 | 6.4±0.6 |
|  | E49C^Alexa594^ | 0.8±0.1 | 7.1±0.9 |
|  | Y274W-N70C^CPM^ | 0.60±0.06 | 5.7±0.6 |
|  | Y274W-K26C^CPM^ | 0.9±0.2 | 6.5±0.8 |
| Palm | S96C | 0.9±0.1 | 7.1±0.9 |
|  | S112C | 0.63±0.07 | 6.1±0.5 |
|  | N130C | 0.8±0.1 | 6.8±0.8 |
|  | S96C^Alexa594^ | 0.8±0.1 | 6.5±0.9 |
|  | S112C^Alexa594^ | 0.51±0.03 | 5.3±0.2 |
|  | N130C^Alexa594^ | 0.66±0.07 | 6.1±0.7 |
| Thumb | S207C | 0.74±0.09 | 7.7±0.9 |
|  | K172C | 0.7±0.2 | 6±1 |
|  | S207C^Alexa594^ | 0.62±0.06 | 6.5±0.7 |
|  | K172C^Alexa594^ | 0.44±0.03 | 4.1±0.6 |
| Little Finger | K329C | 0.50±0.06 | 5.1±0.6 |
|  | R267C | 0.57±0.06 | 5.8±0.7 |
|  | K329C^Alexa594^ | 0.44±0.04 | 4.9±0.6 |
|  | R267C^Alexa594^ | 0.57±0.04 | 4.3±0.4 |
| ^a^Obtained from the fits to a single-exponential equation [19,20]: [Product] = A[1 - exp(- *k_obs_*t)]. | | | |
