# Supplementary material for: Global Conformational Dynamics of a Y-Family DNA Polymerase during Catalysis
Source: PLoS Biol. 2009 Oct 27;7(10):e1000225. doi: 10.1371/journal.pbio.1000225 (PMC2758995; doi:10.1371/journal.pbio.1000225)
Supplement: Table S4 — The phase rates (s−1) derived from stopped-flow kinetic assays are the average from multiple independent experiments and are reported as mean ± standard deviation. (0.08 MB DOC) [file pbio.1000225.s010.doc]

| **Table S4.** The phase rates (s^-1^) derived from stopped-flow kinetic assays are the average from multiple independent experiments and are reported as mean ± standard deviation. | | | | | | | | | |
| --- | --- | --- | --- | --- | --- | --- | --- | --- | --- |
| **Domain**  **Mutant**  **(location)** | **DNA** | **Phase Rate** (s^-1^) | | | | | | | |
|  |  | **Donor** | | | | **Acceptor** | | | |
|  |  | **P_1_** | | **P_2_** | | **P_1_** | | **P_2_** | |
|  |  | **20 °C** | **37 °C** | **20 °C** | **37 °C** | **20 °C** | **37 °C** | **20 °C** | **37 °C** |
| Finger  N70C^a^  (loop) | S-1 | 14±2 | 33±5 | 0.53±0.06 | 2.6±0.6 | 13±3 | 27±2 | 0.45±0.09 | 2.6±0.1 |
|  | S-2 | 15±7 | 25±5 |  |  | 22±7 | 23±5 |  |  |
| Finger  E49C^a^  (helix) | S-1 | 19±2 | 36±5 | 0.78±0.09 | 3.7±0.6 | 21±6 | 37±5 | 0.81±0.08 | 3.8±0.7 |
|  | S-2 | 18±2 | 33±1 |  |  | 21±5 | 34±2 |  |  |
| Palm  S96C^a^ (loop) | S-1 | 16±1 | 22±1 | 0.55±0.09 | 3.0±0.6 | 19±2 | 26±5 | 0.53±0.08 | 3.1±0.5 |
|  | S-2 | 18±3 | 28±2 |  |  | 14±2 | 23±2 |  |  |
| Palm  S112C^a^  (loop) | S-1 | 12±1 | 21±2 | 0.62±0.01 | 3.2±0.3 | 13±2 | 20±1 | 0.61±0.03 | 3.1±0.4 |
|  | S-2 | 11±1 | 25±4 |  |  | 12±2 | 23±3 |  |  |
| Palm  N130C^a^  (helix) | S-1 | 16±5 | 24±3 | 0.65±0.09 | 6±2 | 13±5 | 22±1 | 0.73±0.05 | 6±3 |
|  | S-2 | 15±3 | 26±4 |  |  | 14±3 | 28±9 |  |  |
| Thumb  S207C^a^ (loop) | S-1 | 14±2 | 35±2 | 0.52±0.08 | 4±1 | 13±2 | 32±6 | 0.52±0.09 | 3.9±0.5 |
|  | S-2 | 15±5 | 30±3 |  |  | 12±1 | 28±2 |  |  |
| Thumb  K172C^a^  (helix) | S-1 | 11±2 | 22±3 | 0.51±0.03 | 3.2±0.3 | 12±2 | 23±2 | 0.44±0.02 | 3.4±0.2 |
|  | S-2 | 13±1 | 23±6 |  |  | 14±2 | 24±6 |  |  |
| LF  K329C^a^  (loop) | S-1 | 15±1 | 33±6 | 0.51±0.08 | 5.3±0.6 | 18±3 | 30±6 | 0.48±0.03 | 6±1 |
|  | S-2 | 17±5 | 28±6 |  |  | 18±5 | 25±7 |  |  |
| LF  R267C^a^  (helix) | S-1 | 14±2 | 33±6 | 0.5±0.1 | 4.1±0.5 | 13±2 | 35±3 | 0.44±0.01 | 4.0±0.1 |
|  | S-2 | 18±4 | 37±7 |  |  | 18±5 | 34±4 |  |  |
| LF/Finger  N70C^b^ | S-3 |  |  |  |  | 25±6 |  | 0.52±0.02 |  |
|  | S-4 |  |  |  |  | 24±7 |  |  |  |
| LF/Finger  K26C^b^ | S-3 |  |  |  |  | 18±2 |  | 0.44±0.09 |  |
|  | S-4 |  |  |  |  | 15±1 |  |  |  |
| ^a^Each of the Alexa594-labelled mutants contains the C31S mutation and is listed in Table S1.  ^b^Each of the CPM-labelled mutants contains the C31S and Y274W mutations and is listed in Table S2. The rates derived from CPM-labelled mutants are not included in the calculations of average P_1_ and P_2_ rates. | | | | | | | | | |
