# Supplementary material for: Global Conformational Dynamics of a Y-Family DNA Polymerase during Catalysis
Source: PLoS Biol. 2009 Oct 27;7(10):e1000225. doi: 10.1371/journal.pbio.1000225 (PMC2758995; doi:10.1371/journal.pbio.1000225)
Supplement: Table S5 — Measured motion distances of each Alexa594-labelled amino acid residue of Dpo4 during correct nucleotide binding at 20°C using steady-state FRET data. (0.05 MB DOC) [file pbio.1000225.s011.doc]

| **Table S5.** Measured motion distances of each Alexa594-labelled amino acid residue of Dpo4 during correct nucleotide binding at 20 °C using steady-state FRET data. | | | | | |
| --- | --- | --- | --- | --- | --- |
| **Dpo4**  **domain** | **Fluorescently-labelled Dpo4 residue** | **Distance between each FRET pair** (Å)^a^ | | **Net movement of each Dpo4 residue during elementary steps in Figure 7** (Å)^d^ | |
|  |  | **d_1_**^b^ | **d_2_**^c^ | **Steps 2-4**  (d_2_ – d_1_) | **Steps 3-4**^e^ |
| Finger | N70C^Alexa594^ | 60.44 | 61.68 | 1.24 | - 2.54 |
|  | E49C^Alexa594^ | 55.49 | 56.89 | 1.40 | - 1.56 |
| Palm | S96C^Alexa594^ | 71.65 | 72.55 | 0.90 | 1.86 |
|  | S112C^Alexa594^ | 75.23 | 75.21 | - 0.02 | 1.33 |
|  | N130C^Alexa594^ | 55.21 | 56.08 | 0.87 | 0.23 |
| Thumb | S207C^Alexa594^ | 48.91 | 49.35 | 0.44 | 0.45 |
|  | K172C^Alexa594^ | 56.61 | 57.12 | 0.51 | 0.61 |
| Little Finger | K329C^Alexa594^ | 53.85 | 55.37 | 1.52 | - 3.69 |
|  | R267C^Alexa594^ | 50.49 | 51.88 | 1.39 | - 1.96 |
| ^a^Distance between each Alexa594-labelled Dpo4 residue (acceptor) and the Alexa488-labelled 9^th^ primer base (donor) from the 3′-terminus of S-1 DNA (Table 1) was calculated using the following equation: *E*_T_ = 1 - F_AD_/F_D_ = 1/[1 + (r/R_0_)^6^], where *E*_T_: FRET efficiency; F_AD_ and F_D_: the donor peak fluorescence intensity in the presence and absence of an acceptor, respectively; R_0_: the Förster distance (60 Å) of the FRET pair of Alexa488 and Alexa594; and r: calculated distance between the donor and acceptor.  ^b^Distance was calculated based on the change in donor peak fluorescence intensity between the binary complex Dpo4•S-1 and S-1 DNA (Table 1) alone at 20 °C.  ^c^Distance was calculated based on the change in donor peak fluorescence intensity between the ternary complex Dpo4•S-2•dTTP and S-2 DNA (Table 1) alone at 20 °C.  ^d^Positive and negative values indicate that the Dpo4 residue respectively moves away from and towards DNA.  ^e^Predicted residue motions for Steps 3-4 were calculated from the difference between the measured net movements during Steps 2-4 and the predicted net movements during Step 2 in Table S6. | | | | | |
