# Supplementary material for: Global Conformational Dynamics of a Y-Family DNA Polymerase during Catalysis
Source: PLoS Biol. 2009 Oct 27;7(10):e1000225. doi: 10.1371/journal.pbio.1000225 (PMC2758995; doi:10.1371/journal.pbio.1000225)
Supplement: Table S6 — Predicted motion distances of each selected amino acid residue of Dpo4 during correct nucleotide binding on the basis of the binary and ternary crystal structures of Dpo4. (0.05 MB DOC) [file pbio.1000225.s012.doc]

| **Table S6.** Predicted motion distances of each selected amino acid residue of Dpo4 during correct nucleotide binding on the basis of the binary and ternary crystal structures of Dpo4. | | | | | | | |
| --- | --- | --- | --- | --- | --- | --- | --- |
| **Dpo4**  **domain** | **Selected Dpo4 residue** | **Calculated distance**  (Å) | | | **Net movement of each Dpo4 residue during elementary steps in Figure 7** (Å)^d^ | | |
|  |  | **d_1_**^a^ | **d_2_**^b^ | **d_3_**^c^ | **Steps 2-4**  (d_2_ – d_1_) | **Step 2**  (d_3_ – d_1_) | **Steps 3-4**  (d_2_ – d_3_) |
| Finger | N70 | 48.15 | 51.08 | 51.93 | 2.93 | 3.78 | - 0.85 |
|  | E49 | 45.04 | 47.57 | 48.00 | 2.53 | 2.96 | - 0.43 |
| Palm | S96 | 39.05 | 38.49 | 38.09 | - 0.56 | - 0.96 | 0.40 |
|  | S112 | 40.57 | 39.98 | 39.22 | - 0.59 | - 1.35 | 0.76 |
|  | N130 | 47.70 | 48.44 | 48.34 | 0.74 | 0.64 | 0.10 |
| Thumb | S207 | 36.17 | 37.09 | 36.16 | 0.92 | - 0.01 | 0.93 |
|  | K172 | 41.20 | 41.82 | 41.10 | 0.62 | - 0.10 | 0.72 |
| Little Finger | K329 | 23.14 | 27.09 | 28.35 | 3.95 | 5.21 | - 1.26 |
|  | R267 | 25.72 | 27.56 | 29.07 | 1.84 | 3.35 | - 1.51 |
| ^a^Calculated distance between the C_α_ atom of a Dpo4 residue and the C_1_′ atom of the 9^th^ primer base from the 3′-terminus of Dpo4-bound DNA based on the binary crystal structure of Dpo4•DNA (PDB code 2RDJ) in [8].  ^b^Calculated distance between the C_α_ atom of a Dpo4 residue and the C_1_′ atom of the 9^th^ primer base from the 3′-terminus of Dpo4-bound DNA based on the ternary crystal structure of Dpo4•dideoxyDNA•matched dATP (PDB code 2AGQ) in [36].  ^c^Calculated distance between the C_α_ atom of a Dpo4 residue and the C_1_′ atom of the 10^th^ primer base from the 3′-terminus of Dpo4-bound DNA based on the binary crystal structure of Dpo4•DNA (PDB code 2RDJ) in [8].  ^d^Positive and negative values indicate that the Dpo4 residue respectively moves away from and towards DNA. | | | | | | | |
